# Research Progress on Responses and Regulatory Mechanisms of Plants Under High Temperature

**DOI:** 10.3390/cimb47080601

**Published:** 2025-08-01

**Authors:** Jinling Wang, Yaling Wang, Hetian Jin, Yingzi Yu, Kai Mu, Yongxiang Kang

**Affiliations:** 1 College of Forestry, Northwest A&F University, Yangling 712100, China; wjlbhdx@nwafu.edu.cn; 2 Engineering Research Centre of Forestry Biotechnology of Jilin Province, College of Forestry, Beihua University, Jilin 132013, China; 3 Xi’an Botanical Garden of Shaanxi, Xi’an 710061, China; wangyaling@hainanu.edu.cn

**Keywords:** high temperature, physiology and biochemistry, regulatory network, heat resistance

## Abstract

Global warming has resulted in an increase in the frequency of extreme high-temperature events. High temperatures can increase cell membrane permeability, elevate levels of osmotic adjustment substances, reduce photosynthetic capacity, impair plant growth and development, and even result in plant death. Under high-temperature stress, plants mitigate damage through physiological and biochemical adjustments, heat signal transduction, the regulation of transcription factors, and the synthesis of heat shock proteins. However, different plants exhibit varying regulatory abilities and temperature tolerances. Investigating the heat-resistance and regulatory mechanisms of plants can facilitate the development of heat-resistant varieties for plant genetic breeding and landscaping applications. This paper presents a systematic review of plant physiological and biochemical responses, regulatory substances, signal transduction pathways, molecular mechanisms—including the regulation of heat shock transcription factors and heat shock proteins—and the role of plant hormones under high-temperature stress. The study constructed a molecular regulatory network encompassing Ca^2+^ signaling, plant hormone pathways, and heat shock transcription factors, and it systematically elucidated the mechanisms underlying the enhancement of plant thermotolerance, thereby providing a scientific foundation for the development of heat-resistant plant varieties.

## 1. Introduction

Temperature plays a crucial role in various plant physiological and developmental processes, including seed germination, vegetative growth, morphological structure development, and floral organ formation. Plant growth depends on an appropriate temperature range, with most species exhibiting optimal growth within a temperature range of 0 °C to 30 °C [1]. Different species demonstrate distinct adaptive capacities to temperature variations, and exceeding their optimal temperature thresholds can negatively impact plant growth and development. Due to global warming, global temperatures are on the rise, and this warming trend is also evident across various regions of China. In recent years, temperatures reaching 40 °C and above have increasingly occurred across various regions of the country, adversely affecting the normal growth and development of plants. High temperatures can compromise the stability of plant cell membranes, inducing physiological disorders and negatively impacting nutrient metabolism, photosynthesis, respiration, and other essential physiological processes [2,3,4]. Unlike animals, plants are unable to actively avoid exposure to high temperatures; however, they have evolved unique defense mechanisms to cope with such environmental stress. Upon exposure to high temperatures, plants enhance their antioxidant capacity and mitigate damage caused by elevated temperatures through a range of physiological and biochemical responses, cellular signal transduction pathways, and gene regulatory mechanisms.

## 2. Morphological and Leaf Anatomical Structural Alterations in Plants Under High-Temperature Stress

Leaves are vital organs responsible for photosynthesis, respiration, and transpiration in plants, and they demonstrate a more immediate and observable response to elevated temperatures. Therefore, leaves are widely regarded as the primary organs for studying heat resistance. Under high-temperature stress, leaves progressively exhibit various symptoms, including shrinkage, curling, marginal scorching, browning, and, in severe cases, desiccation. Moreover, newly emerged leaves are more prone to shrinkage and curling than mature leaves [5,6].

At room temperature, the cellular structures of leaves, such as the cell membrane, chloroplasts, mitochondria, and nucleus, exhibited a normal morphology. The intercellular spaces between plant cells were comparatively narrow. Chloroplasts displayed an elongated shape and were arranged adjacent to the cell wall. The grana were arranged in an orderly manner, and the plasma membrane, vacuolar membrane, and nuclear membrane were clearly distinguishable [7,8]. Under high-temperature conditions, the mesophyll cells underwent significant structural alterations, and plasmolysis became increasingly pronounced [9]. The plasma membrane and vacuolar membrane were disrupted, while the chloroplast membrane was either partially or even completely disintegrated. The thylakoid lamellae appeared loosely and haphazardly arranged, and the chloroplasts exhibited enlargement. Some displayed distorted and irregular shapes, gradually migrating toward the central region of the cell [7,8]. The spongy mesophyll tissue in the leaves was significantly shrunken, and the palisade mesophyll cells also showed signs of shrinkage, making the distinct palisade arrangement almost unrecognizable [10].

## 3. Effects of High Temperature on Plant Physiology and Biochemistry

The impacts of high temperature on plant physiology and biochemistry have consistently been a focal point of scientific research. Recently, studies on the effects of high-temperature stress on plants have expanded from model species (such as rice and *Arabidopsis*) [11,12] to a broader range of plant taxa [13,14,15]. The response mechanisms of various plants to high-temperature environments are increasingly being elucidated, offering valuable insights into the understanding of plant heat-tolerance mechanisms and providing a theoretical foundation for the development of heat-resistant crop varieties.

### 3.1. Effects of High-Temperature Stress on Photosynthesis

Generally, plant photosynthesis begins to decline when temperatures exceed 35 °C and typically ceases entirely within the temperature range of 40 °C to 50 °C. High-temperature stress can damage the structure of chloroplasts, mitochondria, and cytoplasm, degrade photosynthetic pigments, reduce chlorophyll content [16], inhibit photosynthesis, and, thereby, disrupt the normal functioning of plant photosynthetic processes. Under different high-temperature conditions, the factors contributing to the reduction in photosynthetic capacity varied. When the temperature ranged from 35 °C to 40 °C, the inhibition of photosynthesis was primarily attributed to stomatal limitations. However, when the temperature exceeded 40 °C, the decline in photosynthetic capacity was mainly due to non-stomatal factors [17,18]. However, in the study of maize net photosynthetic rate, it was observed that the net photosynthetic rate began to decline when leaf temperature exceeded 38 °C, and this reduction was not attributed to stomatal responses under high-temperature conditions [19].

Under high-temperature conditions, stomatal conductance (*Gs*) varies among different plant species. When the temperature increased from 30 °C to 40 °C, the stomatal conductance of *Populus deltoides* × *nigra* and *Pinus taeda* increases by 42% and 40%, respectively [20]. High-temperature stress led to a decrease in both the net photosynthetic rate (*Pn*) and stomatal conductance (*Gs*) in the leaves of kiwifruit [21] and *Ficusconcinna* var. *Subsessilis* [22]. Under high-temperature stress, the transpiration rate of plants exhibits corresponding changes. High temperature was observed to decrease the transpiration rate (*Tr*) of kiwifruit [21] and sesame leaves [23], whereas in *Ficusconcinna* var. *Subsessilis* [22] and grape [24], the transpiration rate initially increased rapidly under high-temperature conditions and subsequently stabilized.

Photosystem II (PSII) is highly sensitive to high temperatures and prone to damage [25]. Elevated temperatures impair the electron transport capacity of PSII. Under such conditions, the photochemical reactions in plant leaves are inhibited, resulting in reduced light energy absorption, quantum yield, and electron transfer efficiency of PSII. The maximal photochemical efficiency (Fv/Fm) exhibited a downward trend. High temperatures impeded energy transfer within the central thylakoids of PSII, resulting in irreversible inactivation [26,27]. The decrease in Fv/Fm in the heat-sensitive strain was significantly greater than that in the heat-resistant strain, indicating its potential as a marker for heat resistance [28,29].

Under short-term high-temperature stress (45 °C to 50 °C), the maximum photochemical efficiency of PSII (Fv/Fm) in *Manglietia aromatica* Dandy, *Manglietia megaphylla* Hu et Cheng, and *Manglietia grandis* Hu et Cheng decreased significantly, whereas the initial fluorescence (F0) increased [30]. However, certain Magnoliaceae species, such as *Manglietiastrum sinicum*, *Magnolia biloba*, *Tsoongiodendron odorum*, and *Parakmeria yunnanensis*, demonstrate strong adaptability to high-temperature environments and can be successfully introduced into regions with an average annual temperature of 20.6 °C and extreme maximum temperatures reaching up to 42 °C [31].

### 3.2. Effect of High Temperature on Regulatory Substances in Plants

Some osmolytes maintain physiological functions, facilitate osmoregulation, and protect subcellular structures. Moreover, certain osmolytes are capable of scavenging reactive oxygen species (ROS). Under high-temperature conditions, the levels of soluble sugars, proline (Pro), and malondialdehyde (MDA) in plants change dynamically to help regulate the plant’s response to temperature stress.

Under high-temperature conditions, the levels of malondialdehyde (MDA), proline (Pro), soluble sugars, soluble proteins, and electrolyte leakage (EL) in plants increased with rising temperatures [32,33,34]. However, there are differences among various plant varieties. Generally, heat-tolerant varieties exhibit higher proline content compared to heat-sensitive varieties [35]. When plants are subjected to high temperatures, osmotic substances play a role in regulating the plant defense system. However, as temperature rises, not all regulatory substances exhibit a corresponding increase. For instance, the soluble protein content in rose leaves was observed to initially increase and subsequently decrease with escalating temperatures [34]. This suggests that different plant species may employ distinct mechanisms in response to high-temperature stress.

## 4. Signal Transduction Under High-Temperature Conditions

As a key second messenger in cell signal transduction, Ca^2+^ plays essential roles in maintaining the structural integrity and physiological functions of plant cells. It contributes to the stabilization of the cell wall, cell membrane, and membrane-associated proteins [36], and is involved in the regulation of numerous physiological and biochemical processes in plants. Ca^2+^ are typically stored in various intracellular and extracellular organelles and are maintained at low concentrations [37,38]. When plants are subjected to high-temperature stress, the Ca^2+^ channel opens, resulting in an elevated intracellular Ca^2+^ concentration. This increase enables Ca^2+^ to bind with calmodulin (CaM), forming a Ca^2+^-CaM complex that activates specific target enzymes or proteins [39]. Consequently, this activation regulates various cellular processes, such as transcription, protein phosphorylation, and metabolic modifications, ultimately triggering appropriate physiological and biochemical responses. When the calcium signal has fulfilled its physiological role, cytosolic Ca^2+^ is transported out of the cell primarily through the plasma membrane Ca^2+^ ATPase (PMCA) and the Na^+^–Ca^2+^ exchanger (NCX). In parallel, Ca^2+^ can be resequestered into the endoplasmic reticulum via the sarco/endoplasmic reticulum Ca^2+^ ATPase (SERCA), which effectively restores the cytosolic Ca^2+^ concentration to its resting state level [40,41].

When exogenous Ca^2+^ levels increased, the damage caused by high temperatures to plants was alleviated, with improvements observed in chlorophyll content, total soluble protein levels, and antioxidant enzyme activity [42,43]. High temperature causes an influx of Ca^2+^, initiating downstream heat stress (HS) signaling pathways. At high temperatures, Ca^2+^ treatment triggers a significant and prolonged elevation of intracellular Ca^2+^ levels, thereby promoting the expression of rice CaM1–1 isoforms and nuclear small heat shock protein genes (sHSPC/N). Furthermore, the transient elevation of intracellular Ca^2+^ triggered by heat stress (HS) can be physiologically modulated in accordance with the intensity of the HS stimulus [44]. Elevated Ca^2+^ levels not only activate the calcium sensor calmodulin (CaM) but also trigger the activation of calcium-dependent protein kinases (CDPKs) and calcium/calmodulin-dependent protein kinases (CaMKs). Ca^2+^ can modulate the expression of stress-responsive genes by regulating the phosphorylation and dephosphorylation of specific transcription factors through the action of certain phosphatases [45]. *Arabidopsis thaliana* Calcium/Calmodulin-binding Kinase 3 (AtCBK3) phosphorylates heat shock factor AtHsfA1a to control the binding activity of heat shock factors (HSFs) to heat shock elements (HSEs), thereby regulating the transcription of heat shock protein (HSP) genes and the synthesis of HSPs [46]. CaM interacts with protein phosphatase 7 (PP7) to modulate HSF activity, thereby activating the expression of heat shock protein (HSP) genes. The expression levels of AtHSP70 and AtHSP101 were significantly upregulated in AtPP7 overexpression lines after heat shock treatment. These findings suggest that AtPP7 may play a role in regulating HSP gene expression and enhancing plant thermotolerance [47].

The Calmodulin-like (CML) gene family constitutes a plant-specific group of calcium sensors. When expressed at high levels in *Arabidopsis*, SlCML39—a CML gene isolated from tomato—exhibits inhibitory effects on germination rates and seedling growth under high-temperature-stress conditions. These findings suggest that SlCML39 plays a regulatory role in plant responses to heat stress [48]. The cyclic nucleotide-gated calcium channel (CNGC) is involved in the mechanism underlying the plant heat-stress response. Loss of function of CNGC2/CNGCb increases plant tolerance to heat stress, indicating that CNGC2/CNGCb negatively regulates plant thermotolerance [49].

## 5. Molecular Mechanism of High-Temperature Stress

### 5.1. Heat Shock Transcription Factor Regulation

The impact of temperature increase on plants extends beyond physiological effects to encompass molecular-level changes within the organism. When exposed to high temperatures, plants activate a series of stress responses, including the expression of relevant genes, the accumulation of transcriptional regulators, and the activation of heat shock proteins (HSPs), which help protect plants from damage or mitigate its severity [50,51,52,53,54].

After heat-stress treatment, the expression levels of ZmHsf and ZmHsp70 in maize were significantly upregulated, which contributed to the regulation of maize’s heat tolerance [55]. ZmHsf 23L and ZmHsf 23S are significantly upregulated under heat-stress conditions. Mutants deficient in ZmHsf23L or lacking both ZmHsf 23L and ZmHsf 23S exhibit increased sensitivity to heat stress. In contrast, the overexpression of ZmHsf 23S enhances heat tolerance in maize. Furthermore, the co-overexpression of ZmHsf 23L and ZmHsf 23S synergistically improves thermotolerance in transgenic plants [56].

The heat shock transcription factor HSFA1s is widely recognized as a central regulator of plant thermotolerance. Acting upstream of other key transcription factors such as HSFA2, HSFA7a/b, and DREB2A, HSFA1s plays a pivotal role in modulating the plant heat-stress response (HSR) [57]. Under high-temperature conditions, the rice heat shock transcription factor OsHsfA2b is markedly induced and expressed. Compared to the wild type, transgenic rice overexpressing OsHsfA2b exhibits significantly enhanced thermotolerance, characterized by reduced plant damage and an increased survival rate [58].

PeHSFA2 in *Populus euphratica* can significantly enhance plant heat tolerance by regulating the expression of PtoHSP19.9, PtoHSP21.3, PtoHSP21.8, and PtoHSP22.0 in *Populus tomentosa*. This gene exhibits considerable potential for the development of poplar varieties with markedly improved thermotolerance [59]. The expression levels of AtMBF1c, AtZAT12, AtAPX1, AtHSA32, and AtHSPs was significantly upregulated in PtHSFA4a transgenic plants of *Populus trichocarpa*. Moreover, PtHSFA4a directly binds to the promoters of AtAPX1 and AtHSPs under heat-stress conditions, thereby enhancing the heat resistance of leaves by upregulating the antioxidant defense system and maintaining protein-folding homeostasis in leaves [60]. Heat shock transcription factors (HSFs) regulate plant heat tolerance either individually or through interactions, and LlHSFC2 interacts with LlHSFA1, LlHSFA2, and LlHSFA3A. The overexpression of LlHSFA3A alone confers heat tolerance in lilies, whereas the co-overexpression of LlHSFC2 and LlHSFA3A further enhances the heat tolerance of transgenic plants [61].

### 5.2. Heat Shock Protein Regulation

When the ambient temperature exceeds the optimal range for normal plant growth, heat shock proteins (HSPs) are induced as a protective response in plants. In higher plants, short-term exposure to temperatures within the range of 38 °C to 40 °C is sufficient to induce the production of heat shock proteins (HSPs) [62]. Heat shock proteins (HSPs) are classified into five major families based on their molecular weight: HSP100, HSP90, HSP70, HSP60, and small heat shock proteins (sHSPs) [63]. The roles of various heat shock proteins in plants under high-temperature stress are becoming increasingly clear.

The expression of 14 heat shock protein (Hsp) genes in rice was upregulated under high-temperature conditions. Under natural high-temperature environments, the reduction in yield observed in knockout mutants of these 8 Hsp genes was primarily attributed to their effects on seed setting rate or grain weight [64]. HSP90 is a crucial member of the heat shock protein (HSP) family. It not only activates the expression of HSFA to enhance plant thermotolerance [65] but also participates in the high-temperature stress response mechanism in *Arabidopsis thaliana* through functional pathways such as ABA and Ca^2+^ signaling [66]. HSP90 can also actively regulate thermotolerance in tomatoes by activating the expression of other transcription factors [67]. In addition to HSP90, other heat shock proteins play a critical role in enabling cellular adaptation to heat shock.

Mutant plants in *Arabidopsis*, maize, and rice that lack the ClpB protein show increased sensitivity to heat stress compared to wild-type plants [50], and ClpB1-overexpressing transgenic tobacco exhibits significantly enhanced heat tolerance [68]. The HSP70/HSP40 molecular chaperone system plays a critical role in protecting plants from prolonged exposure to heat stress [52]. By modulating photosynthesis-related proteins, sHSPs provide a certain degree of protection to chloroplasts, thereby enhancing the heat tolerance of maize chloroplasts [69]. Overexpression of the HSP17.4 gene significantly improves the heat tolerance of soybean plants [70]. ZmHsf23S enhances thermotolerance by directly promoting the transcription of Hsp16.9, Hsp17.2, and Hsp18a [57].

There is a synergistic interaction among various heat shock proteins. Protein aggregates can be efficiently resolubilized by HSP100/Clp family chaperones and subsequently refolded with the assistance of the Hsp70 system; the final refolding of solubilized proteins into their native forms may be completed by members of the Hsp60 family (GroEL–GroES) [71]. The overexpression of PsHSP70b in *Chlamydomonas* not only enhanced its survival rate but also upregulated the transcription levels of HSF1, CrHSP20, and CrHSP70 genes [72].

## 6. Response of Plant Hormones to High Temperature

Under high-temperature stress, plant hormones can mitigate or enhance resistance to the adverse effects of high temperature on plants. The differential hormone metabolites in Loquat (*Eriobotrya japonica*) fruits under 40 °C stress were investigated. It was found that 37 hormone metabolites were differentially enriched in the fruits under high-temperature stress, including auxins, jasmonic acid, abscisic acid, salicylic acid, ethylene, and cytokinin [73].

### 6.1. Auxin

Auxins is a plant hormone that not only promotes growth but also plays a critical role in the plant’s response to heat stress. A total of 58 differentially expressed genes (DEGs) related to auxin signaling were identified in fruits exposed to high-temperature stress, and auxin-enriched hormone metabolites constituted 24.32% of the total metabolites [73]. These findings indicate that auxin is involved in the defense mechanisms activated by plants under high-temperature stress conditions.

The expression of the auxins receptor TIR1/AFB2 in rice spikelets was significantly downregulated under high-temperature stress. Exogenous IAA application could reduce membrane lipid peroxidation and ROS accumulation in spikelets [74], thereby alleviating high-temperature-induced damage. Auxins interact with HSP90 in *Arabidopsis thaliana*, which plays a role in regulating temperature-dependent seedling growth by stabilizing the auxin receptor TIR1. When the molecular chaperone HSP90 is present in plants, a slight increase in ambient temperature promotes the rapid accumulation of the auxin co-receptor TIR1. However, the inhibition of HSP90 activity leads to the degradation of TIR1 [75]. The auxin co-receptor TIR1 interacts with HOP proteins and plays a role in plant responses to high-temperature stress. In *Arabidopsis* triple mutants of hop1, hop2, and hop3, exposure to high temperatures inhibited plant growth, reduced auxin sensitivity, decreased TIR1 accumulation, and suppressed auxin-regulated transcriptional responses [76].

### 6.2. Abscisic Acid

Abscisic acid (ABA) is a stress-related hormone that mitigates membrane damage and promotes the accumulation of osmotic substances, thereby enhancing plant adaptability to various environmental stresses. Under high-temperature stress, the biosynthesis of endogenous ABA is upregulated, its degradation is reduced, or its bound form is released, leading to an increase in ABA concentration and improved plant resistance.

Abscisic acid regulates the synthesis of new stress-resistant proteins and enhances stress resistance by inducing the expression of stress-related genes. Additionally, abscisic acid promotes stomatal closure, reducing water loss, maintaining water balance, and minimizing stress damage [77]. ABA induces the expression of catalase (CAT1) through the MAPK signaling cascade mediated by AtMKK1 and AtMPK6, which also promotes H_2_O_2_ production. The overexpression of AtMKK1 and AtMPK6 significantly enhances the ABA-induced responses [78].

ABA can induce the expression of HSF and HSP genes. In tobacco, ABA treatment stimulates the expression of NtHSP70-1, which helps mitigate plant stress responses under adverse conditions [79]. Under high-temperature stress, NtHSP70-8b positively regulates the tobacco response to heat stress. In tobacco plants overexpressing NtHSP70-8b, the expression levels of genes involved in ABA synthesis and signaling pathways (NtNCED3 and NtAREB), stress defense mechanisms (NtERD10C and NtLEA5), and other heat shock proteins (NtHSP90 and NtHSP26a) were significantly upregulated [80]. ABA pretreatment induced the expression of heat-shock-related genes (OsHSP23.7, OsHSP17.7, OsHSF7, and OsHsfA2a), reduced cellular damage, and enhanced antioxidant defense capability [81]. Furthermore, ABA serves as an inducer of the transcription factors ERF74 and ERF75 under high-temperature conditions. These transcription factors form a complex regulatory network that modulates plant heat tolerance [82].

The synergistic effect of ABA and NO mitigated the damage caused by high-temperature stress in plants, decreased MDA content, and enhanced the activity of antioxidant enzymes. Exogenous ABA and NO treatment significantly alleviated these effects. Moreover, the ability of ABA to alleviate heat stress increased with rising levels of NO [83,84].

### 6.3. Ethylene

Ethylene (ET), a plant hormone, plays a critical role in regulating plant growth, development, and the response to heat stress. Under normal conditions, endogenous ethylene levels are maintained at a low concentration, allowing the ethylene receptor to remain active and sustain the kinase CTR1 in its active state, thereby suppressing ethylene signaling. However, upon exposure to high-temperature stress, ethylene biosynthesis is enhanced, leading to an increase in ethylene accumulation within the plant [73]. The membrane-anchored receptor family detects ethylene, and ligand binding leads to receptor inactivation, which subsequently inactivates CTR1 and activates EIN2. This process initiates a transcriptional cascade in which the EIN3/EIL and ERF transcription factors function in a sequential manner [85]. It then initiates the regulation of downstream genes in response to stress. Under high-temperature stress, the activities of ACS and ACO—key enzymes involved in ethylene synthesis—in the leaves of *Rhododendron* increase, leading to a higher release rate of endogenous ethylene. This, in turn, affects the heat resistance of *Rhododendron* by modulating the antioxidant system [86]. The basal thermotolerance of ethylene signaling-deficient mutants is reduced [82,87]. The AP2/ERF family transcription factors associated with ethylene response were upregulated following high-temperature stress [88]. Ethylene can also induce photosynthetically derived sugars, enhance photosynthesis and metabolism, and, thereby, promote plant growth and stress resistance [89].

### 6.4. Brassinosteroids

Brassinosteroids (BRs), one of the six major classes of plant hormones, function not only in promoting plant growth but also in enhancing stress resistance as key regulators of abiotic and biotic stress responses. The most well-known brassinosteroids include brassinolide (BL), castasterone (CS), and 24-epibrassinolide (EBL), among others.

Brassinosteroids exert their biological effects through signal transduction pathways. Specifically, brassinosteroids bind to the cell surface receptor kinase BRI1, which subsequently phosphorylates and activates two key downstream BR signaling kinases: brassinosteroid signaling kinase 1 (BSK1) and constitutive differential growth 1 (CDG1). This subsequently triggers the phosphorylation and activation of the protein serine/threonine phosphatase BRI1-Suppressor 1 (BSU1), thereby enhancing its enzymatic activity. Activated BSU1 dephosphorylates brassinosteroid-insensitive 2 (BIN2), which serves as a central negative regulator of BR signaling [90]. BIN2, located upstream of HsfA1d, attenuates plant heat tolerance by inhibiting the function of the HsfA1d protein. The removal of BIN2′s inhibitory effect facilitates the nuclear localization and DNA-binding activity of HsfA1d, thereby enhancing plant thermotolerance [91].

When the BR signal is perceived, the plasma membrane-associated NADPH oxidase (RBOH) is activated, leading to increased H_2_O_2_ production and enhanced enzymatic activity [92], thereby improving plant heat tolerance. MAP4K4/TOT3 (Mitogen-activated Protein Kinase Kinase Kinase Kinase 4/Target of Temperature 3) modulates the high-temperature stress response in *Arabidopsis* and wheat via BZR1-mediated signaling pathways [93]. EBR treatment improved the basal heat tolerance of plants. Under high-temperature stress, the exogenous application of brassinolide decreased MDA content while increasing the levels of soluble proteins and soluble sugars, along with enhancing SOD and CAT activities, thereby contributing to improved heat tolerance in Avena nuda [94]. The net CO_2_ assimilation rate, the maximum carboxylation rate of Rubisco, and the quantum yield of PSII electron transport were markedly increased by EBR application. Furthermore, the contents of sucrose, soluble sugars, and starch were significantly elevated, indicating that EBR enhances the CO_2_ assimilation capacity through promoting the Calvin cycle activity [95].

### 6.5. Jasmonic Acid

Jasmonic acid (JA) is a newly recognized type of endogenous plant hormone that plays a crucial role in regulating plant growth, development [96,97,98], and defense responses [99,100]. Jasmonic acid (JA) is synthesized from α-linolenic acid, which is released from the chloroplast membrane and serves as the initial substrate. The biosynthetic pathway of JA involves a series of sequential enzymatic reactions catalyzed by lipoxygenase (LOX), allene oxide synthase (AOS), allene oxide cyclase (AOC), and 12-oxophytodienoate reductase (OPR3). This is followed by three cycles of β-oxidation, ultimately leading to the formation of JA [101].

When plants are exposed to high temperatures, the synthesis of JA is induced, leading to the formation of the JA-Ile complex. This complex subsequently binds to the receptor coronatine-insensitive 1 (COI1). COI1 interacts with JAZ proteins, leading to their ubiquitination by the E3 ubiquitin ligase SCFCOI1 (Skp1/Cullin1/F-box) complex. The ubiquitinated JAZ proteins are subsequently recognized and degraded by the 26S proteasome, thereby releasing downstream transcription factors such as MYC, MYB, and NAC [102,103]. In turn, it regulates the transcription of JA response genes and activates the defense mechanisms in plants against external stress.

Under high-temperature stress conditions, OPR3, a key enzyme in JA synthesis, is activated, which promotes JA biosynthesis and subsequently activates the JA signaling pathway. Consequently, the expression of DREB2A is upregulated [104], leading to the regulation of plant responses to high temperatures, alleviation of heat-induced damage, and enhancement of plant thermotolerance.

### 6.6. Salicylic Acid

Salicylic acid (SA) is an endogenous plant hormone that is widely present in higher plants. It plays a crucial role not only in regulating plant growth but also in mediating the plant’s response to high-temperature stress. The two primary metabolic pathways involved in SA biosynthesis are the isochorismate synthase pathway (ICS pathway) and the phenylalanine ammonia-lyase pathway (PAL pathway) [105]. The PAL pathway plays a crucial role in the biosynthesis of SA and also serves as a key regulator of the steady-state stomatal aperture, thereby enhancing plant adaptability under stress conditions [106].

Exogenous SA can significantly alleviate heat-induced damage by reducing the heat injury index, decreasing electrolyte leakage and malondialdehyde content, and mitigating chlorophyll degradation in seedlings [107]. It increased the proline and soluble protein content, enhanced protein kinase activity, promoted protein phosphorylation, improved photosynthetic efficiency, elevated the activities of antioxidant enzymes (SOD, POD, CAT, and APX), reduced reactive oxygen species (ROS)-induced damage in plants, and alleviated the adverse effects of high-temperature stress [108,109]. Currently, salicylic acid has been extensively utilized as an effective approach to mitigate the adverse effects of high temperature on plant growth and development [110].

## 7. Regulatory Network for Plant Responses to High Temperature

**Figure 1:**
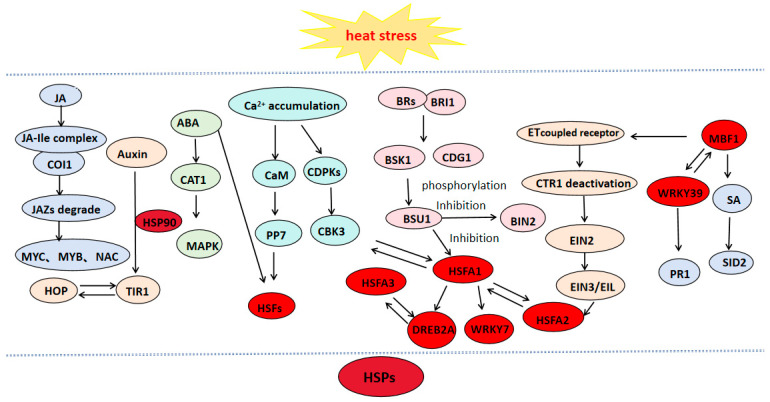
Regulatory network for plant responses to high temperature.

When plants perceive elevated temperatures, calcium influx activates multiple regulatory pathways to coordinate the response to heat stress. These pathways interact synergistically, forming a complex signaling network that modulates plant responses to high-temperature conditions. JA, SA, ethylene, and abscisic acid have been shown to alleviate heat-induced damage in plants and improve their thermotolerance. Transcription factors such as HSF [111], DREB [112], MYB [113], WRKY [114], and MBF1C [115] play an important role in different metabolic pathways and play a role of connection and hub [116,117], for example, MBF1c acts upstream on SA and ethylene during heat stress [118]. Under high-temperature conditions, the Ca^2+^ regulatory network interacts with plant hormone signaling pathways and heat shock transcription factors to establish a comprehensive regulatory network. This network activates the expression of heat shock proteins (HSPs), thereby enhancing the thermotolerance of plants (Figure 1).

## 8. Strategies for Enhancing the Heat Tolerance of Plants

Climate change has resulted in an increasing number of plants being exposed to high temperatures, which severely affects their growth and survival. Under such conditions, plants can mitigate the adverse effects of high temperatures through self-regulatory mechanisms, demonstrating a survival strategy that involves adaptive responses to high temperatures [1]. However, the regulatory capacity of different plants in response to high-temperature damage varies, and certain strategies can be employed to alleviate the adverse effects of high temperatures on plants.

### 8.1. Effects of Heat Acclimation on Plant Responses to High-Temperature Stress

Different plant species exhibit varying responses to high temperatures. Plant heat tolerance is shaped by long-term interactions between plants and their environment throughout the evolutionary process. Through heat acclimation, plants can adjust their physiological and biochemical processes to enter an adaptive state, which enhances their capacity to withstand subsequent high temperatures, thereby enabling normal growth under such conditions.

Özlem Arslan demonstrated that heat acclimation mitigated the adverse effects of high-temperature stress on water content, chlorophyll and carotenoid levels, membrane integrity, photosynthetic efficiency, and the antioxidant defense system in *Cicer arietinum* L. Seedlings acclimated to high temperatures exhibited enhanced thermotolerance, as indicated by improved chlorophyll fluorescence parameters, which suggest a greater capacity to effectively manage the biochemical and physiological changes associated with heat stress [119]. Under high-temperature stress, the degree of decline in the relative water content (RWC) and the ultrastructural damage to chloroplasts in leaves subjected to heat acclimation was lower compared to non-acclimated leaves. Heat-acclimated leaves maintained higher membrane thermal stability, while exhibiting reduced lipid peroxidation as indicated by lower MDA content. Consequently, heat acclimation enhanced the heat resistance of the leaves [120].

This phenomenon of enhanced plant heat resistance through heat acclimation has also been observed in woody species. In *Rhododendron hainanense*, heat acclimation resulted in reduced damage to phenotypic traits, net photosynthetic rate, and membrane stability when compared to non-acclimated plants, thereby improving its tolerance to high temperatures. Furthermore, integrated omics analysis revealed that genes associated with photosynthesis were significantly enriched in heat-acclimated plants relative to their non-acclimated counterparts [121].

### 8.2. Application of Exogenous Substances to Enhance Plant Thermotolerance

The exogenous application of plant hormones and CaCl_2_ [42] as heat-resistant agents can significantly enhance plant heat tolerance. Commonly utilized plant hormones include abscisic acid (ABA) [84], salicylic acid (SA) [108], brassinolide (BR) [122], and ethylene (ET) [86], among others.

Rice seedlings were pretreated with sodium hyposilicate solution (Na_2_SiO_3_·9H_2_O), salicylic acid (SA) solution, calcium chloride solution (CaCl_2_·5H_2_O), and potassium dihydrogen phosphate (KH_2_PO_4_) solution before being exposed to high-temperature stress. The results demonstrated that the application of these four chemical agents significantly enhanced chlorophyll content, superoxide dismutase (SOD), peroxidase (POD), catalase (CAT) activity, and soluble protein levels in rice leaves, while effectively reducing malondialdehyde (MDA) content. This mitigated the adverse effects of high-temperature stress on rice leaves [123]. Under high-temperature stress conditions, the application of heat-resistant agents can effectively alleviate the decline in Fv/Fm values, thereby reducing damage to photosystem II (PSII), suppressing the rapid accumulation of malondialdehyde (MDA) in leaves, promoting the synthesis of free proline (Pro), decreasing the heat injury index, and, ultimately, enhancing the thermotolerance of *Rhododendron* [124,125].

## 9. Conclusions and Future Perspectives

Global warming is an undeniable reality in the present era. The frequency of extreme high-temperature events is increasing, which seriously affects the growth and development of plants. High temperature has become a critical factor constraining plant growth and development. High-temperature stress disrupts cell membrane integrity, decreases photosynthetic efficiency, accelerates chlorophyll degradation, and, ultimately, results in leaf browning, wilting, and even plant desiccation. When plants are subjected to heat stress, a Ca^2+^ influx across the cell membrane is triggered, activating the Ca^2+^ signaling pathway. This activation triggers the plant hormone regulatory pathway, leading to a significant accumulation of plant hormones. Subsequently, various transcription factors interact to form a regulatory network that activates the expression of heat shock proteins (HSPs). Together, these processes mitigate the detrimental effects of high temperatures on plants. At present, the research scope of the mechanism of the effect of high temperature on plants is expanding. Future research should focus more intensively on elucidating the regulatory network underlying the interactions among signal transduction, plant hormones, and heat shock transcription factors under high-temperature conditions, which regulates the heat tolerance mechanisms in plants. Additionally, future research should systematically and deeply explore the mystery of plants’ response to high temperature. Screening heat-resistant plant varieties can facilitate improvements in crop yield and broaden the selection range for landscaping tree species. Utilizing biotechnology to enhance plants’ adaptability to high-temperature stress provides a robust theoretical foundation for genetic breeding.
